# An Algorithm for Mining the Living Habits of Elderly People Living Alone Based on AIoT

**DOI:** 10.3390/s25072299

**Published:** 2025-04-04

**Authors:** Jiaxuan Wu, Yuxin Lu, Yueqiu Jiang

**Affiliations:** School of Information Science and Engineering, Shenyang Ligong University, No. 6, Nanping Central Road, Hunnan New District, Shenyang 110159, China

**Keywords:** elderly people living alone, deep learning, activity recognition, habit mining, association rules

## Abstract

With the global aging population on the rise, the health and safety of elderly individuals living alone have become increasingly critical. This study introduces a novel AIoT-based habit mining algorithm designed to enhance activity monitoring in smart home environments. The proposed method integrates a one-dimensional U-Net neural network for accurate behavioral classification and an FP-Growth-based temporal association rule analysis for uncovering meaningful living patterns. By leveraging environmental sensor data, the algorithm first classifies daily activities and then uses timestamps to detect time-sensitive dependencies in behavior sequences, identifying the long-term habits of the elderly. Experimental validation on CASAS datasets (ARUBA and MILAN) demonstrates superior performance, achieving a precision of 84.77%. Compared to traditional techniques, this approach excels in behavior recognition and habit mining, offering a precise and adaptive framework for AIoT-driven smart home safety and health monitoring systems. The results highlight its potential to improve the quality of life and safety for elderly individuals living alone.

## 1. Introduction

Elderly individuals living alone are often the least able to cope with risks, facing a range of challenges such as health issues, home safety, and difficulties in responding to urgent issues [1,2,3,4,5,6]. At present, the number of elderly people living alone is rising sharply, making their care an increasingly urgent social concern. The latest advancements in IoT hardware, particularly in terms of energy consumption, cost, and interoperability, have driven the development of smart homes [7]. Habit mining algorithms, a data mining technique, can identify frequent patterns from large volumes of user behavior data, revealing the underlying needs and behavioral habits. Considering that the daily activities of elderly individuals at home often exhibit certain correlations and regularities, combining IoT technology with data mining techniques to more accurately identify their potential living habits can not only enhance the quality of life for seniors but also improve their safety at home.

Traditional data mining techniques appear to be incapable of handling large-scale and multi-dimensional smart home data, and they are unable to meet the current high requirements for real-time accuracy and scalability. For example, traditional association rule analysis methods often ignore the time-series characteristics of user behavior when dealing with smart home data, resulting in a lack of timeliness and accuracy in the mined habit patterns. Kong Yinghui et al. used the Apriori algorithm to mine user life behavior patterns [8] and generate the corresponding control strategies accordingly. The associative manipulation habit rules revealed by this approach, although able to show the basic connections between user manipulation actions, do not fully capture the dynamic characteristics of these actions over time [9]. The limitations of the traditional Apriori algorithm have become increasingly apparent in practical applications. During each iteration, the candidate set must scan the database; for instance, if a frequent itemset contains 10 items, the transaction database needs to be scanned at least 10 times. This process generates an enormous candidate set, posing significant challenges to the algorithm’s runtime efficiency and main memory usage [10]. Although some studies have attempted to enhance mining efficiency through algorithmic improvements, these efforts continue to face obstacles, such as high computational complexity and limited adaptability to dynamically evolving data.

The main contributions of this paper are summarized as follows:

- This study uses a one-dimensional U-Net network to classify the behavioral data of elderly individuals living alone. The encoder applies one-dimensional convolution and pooling to capture local features and reduce data dimensions. The decoder uses upsampling and skip connections to restore high-resolution features, integrating detailed information from the encoder. This approach minimizes information loss. The final classification is performed through the output layer, effectively identifying the daily behavior patterns of the elderly.
- By using temporal association rule mining, the inclusion of timestamps helps capture the time dependency and periodicity of behavior patterns, revealing the long-term habits of elderly individuals. For example, it can identify associations like “waking up at 8 AM” or “taking a walk at 10 AM daily”, focusing on both the behaviors and their timing. This method has solved the problem of traditional association rules being unable to effectively handle the time factor, thus enhancing the analysis precision and practicality of behavioral data.
- Experiments show that this algorithm is superior to the existing sota algorithm in all aspects. Regarding behavior recognition, the average balance accuracy of ARUBA is 1.96% higher than that of the sota algorithm with the ARUBA dataset. In the MILAN dataset, the average weighted F1 value and the average balance accuracy of sota algorithm are improved by 1% and 1.82%, respectively. In terms of association rules, with the same dataset, the precision of the proposed algorithm is improved by 7.98% compared with the existing sota algorithm.

**Figure 1:**
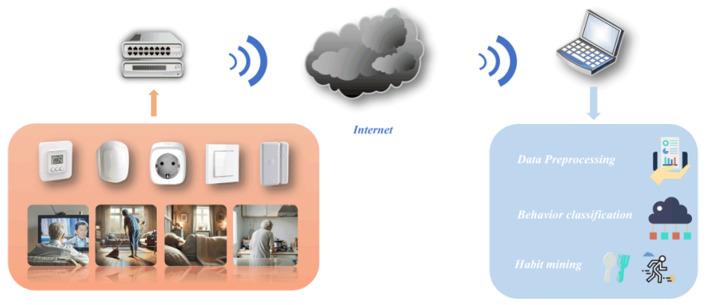
Flowchart of the algorithm.

The overview of the algorithm is shown in Figure 1. Edge sensors collect the life data of the elderly living alone and upload it to the server. Then, the server uploads the collected data to the cloud server. After that, researchers download the data to local devices and then perform tasks such as data preprocessing, behavior classification, and habit mining.

## 2. Related Work

### 2.1. Human Activity Recognition Algorithms

The main task of Human Activity Recognition (HAR) [11,12] is to identify human behaviors from the data collected by various sensors and Internet of Things (IoT) [13,14,15,16,17,18] devices. They utilize different sensor technologies. For example, for the images or videos generated by cameras, technical methods, mainly in the field of computer vision, are used for behavior recognition. For the time-series data produced by wearable devices or low-level intelligent sensors, the traditional methods for dealing with time-series data or machine learning methods will be adopted for analysis.

The Convolutional Neural Network (CNN) [19,20,21,22] is a deep neural network designed specifically for processing image data and is also used for human activity recognition. Ming Zeng et al. [23] applied the CNN to HAR for the first time and built a CNN model based on accelerometer data. Heeryon Cho and Sang Min Yoon [24] further categorized activities into dynamic and static categories and developed CNN models to distinguish them. Prince Fan et al. [25] investigated a new method for human behavior recognition combining a multi-head self-attention mechanism and a temporal convolutional neural network to improve accuracy and reduce computation using knowledge distillation. Dua et al. [26] proposed a multi-input CNN-GRU (Convolutional Neural Network-Gated Recurrent Unit) model based on wearable sensors for human activity recognition. The model combines the spatial feature extraction capability of CNNs and the time series modeling capability of GRUs to improve recognition accuracy. However, CNNs are usually designed for processing image data, and they may not be able to adequately capture information in the temporal dimension when processing time-series data. Although some CNN-based models have been improved in different ways to be applied to human behavior recognition, they may still struggle to accurately classify all behaviors due to the complexity and diversity of activities exhibited by elderly individuals living alone.

The Long Short-Term Memory Network (LSTM) [27,28,29] is a type of deep learning model that can handle sequential data and has also been applied in the Human Activity Recognition (HAR) problem. The LSTM aims to map each window of sensor data to a specific activity. The combination of the Convolutional Neural Network (CNN) and LSTM (known as the ConvLSTM model) [30] takes advantage of the strengths of both architectures to improve performance, and this model is becoming more and more common in dealing with the HAR problem. Francisco Javier Ordóñez proposed a DNN framework called DeepConvLSTM [31], a DNN framework that combines convolutional and recursive layers, and instead of using a pooling layer, models the temporal dynamics of feature maps by using a two-layer LSTM. This model uses CNNs to extract features from raw data, which are then summarized and interpreted by the LSTM. However, the computational complexity of the LSTM model is relatively high, which may lead to long training and inference times when dealing with large-scale behavioral data of elderly people living alone, and it is not conducive to application scenarios with high real-time requirements.

Machine learning methods are also commonly used in human recognition tasks, and the use of decision trees, SVM, and other classifiers [32] can perform this task brilliantly. Zhu, Chung-Bin and Qiu, Hui-Ling [33] proposed a real-time human behavior recognition method based on smartphone sensors, which identifies some universal human behaviors by the sparse locally-preserved projection combined with random forest (SpLPP-RF) method, and it achieves an efficient recognition of behaviors under different cell phone locations. However, machine learning methods are generally more sensitive to noisy and anomalous data, which may negatively affect the training of the model and reduce the generalization ability of the model. Furthermore, they have a limited learning ability for complex behavioral patterns and complex relationships between multimodal data. For example, for some behavior recognition tasks that contain visual, audio, and sensor data at the same time, it is difficult for traditional models to effectively fuse these multimodal information, which affects the recognition accuracy.

### 2.2. Association Rule Algorithms

The main role of association rules [34,35,36,37], as one of the key techniques used in data mining, is to discover valuable or interesting patterns and rules from huge databases, and this algorithm has a wide range of applications in the fields of education, recommender systems, cyber security, and medical diagnosis.

After obtaining the behavioral data of the elderly living alone, the habit mining of correlated behaviors can be carried out with the help of correlation algorithms. In the smart home environment, the research related to correlation habit mining has experienced a certain degree of progress. Sfar, H. et al. used causal association rules to extract the causes of anomalies from a given dataset, which were subsequently used in real-time analysis to detect anomalous risks using a Markov Logic Network machine learning approach, which helps in recommending behaviors that may prevent risk [38]. Kang K. J. et al. [39] demonstrated that useful association rules can be mined even at larger confidence ranges by improving the confidence calculation method. Heierman et al. [40] proposed the Episode Discovery (ED) algorithm for identifying significant events in sequential data and experimentally demonstrated its effectiveness in improving the prediction accuracy of smart home agents. On the other hand, Shaofeng Niu [41] proposed a two-layer ART1 pattern classification method, which combines the Hadoop distributed system and the smart home system to realize the distributed storage and analysis of massive data and solve the problem of insufficient local storage resources. These studies provide powerful data support and control strategies for the design and optimization of smart home systems.

## 3. Proposed Method

This chapter provides a detailed introduction to the design and implementation of the habit mining algorithm for elderly individuals living alone. First, it explains the theoretical foundations of the U-Net network and association rule mining, including their architectural features and working principles. Next, it presents the overall framework of the algorithm, employing a one-dimensional U-Net for behavior classification and integrating FP-Growth [42] for temporal association rule analysis. The data preprocessing section optimizes sensor data handling through techniques such as text-based transformation, index embedding, and a sliding window method. Feature extraction and classification are achieved through the convolutional layers and skip connections of the U-Net, enabling efficient behavior classification. Finally, timestamps are incorporated into association rule mining to enhance the recognition of time-dependent behaviors, uncovering latent patterns in the daily lives of elderly individuals and improving the algorithm’s applicability in smart home environments.

### 3.1. Preliminary

#### 3.1.1. U-Net

The U-Net [43,44,45,46,47] architecture demonstrates significant advantages in data processing, model performance, and practical applications, making it an ideal choice for behavior recognition using sensor time-series data. The U-Net architecture consists of the downsampling and upsampling stages, incorporating only convolutional and pooling layers without fully connected layers. Unlike the Fully Convolutional Network (FCN), U-Net maintains an equal number of convolutional layers in both downsampling and upsampling stages. Moreover, its skip connections directly transfer features from the downsampling layers to the corresponding upsampling layers, ensuring precise pixel localization and high segmentation accuracy, which make it highly effective in semantic segmentation. In reference [48], an FCN was employed as a classifier to classify feature vector sequences, achieving favorable results. Building upon this approach, this study proposes the adoption of the U-Net model for classification tasks on one-dimensional data.

In terms of data processing characteristics, the combination of downsampling and upsampling structures in U-Net, along with convolution, pooling, upsampling, and skip connections, effectively extracts and preserves both local and global features of time-series data. This enables dimensionality reduction while maintaining high-resolution features, which is crucial for capturing the details of behavioral data. Furthermore, U-Net can adapt to the temporal continuity and dynamic variations of behavioral data, aspects that are often challenging for conventional machine learning methods and certain spatial data processing techniques. From a model performance perspective, the integration of multi-level features through skip connections allows U-Net to capture both global and local patterns, thereby achieving the accurate recognition of complex behavioral patterns. It exhibits outstanding performance in analyzing elderly daily activity data, offering high recognition accuracy and strong generalization capability, surpassing shallow neural networks and models prone to overfitting. In practical applications, one-dimensional U-Net demonstrates high computational efficiency, making it suitable for real-time processing on resource-constrained devices. Additionally, its scalability facilitates subsequent adjustments and optimizations for behavior recognition systems, unlike fixed-structure methods that struggle to adapt to such changes.

#### 3.1.2. Association Rule Algorithm

Basket analysis is the initial application of association rules, which can be used to study the purchasing preferences of customers by observing the association between various products they put in their shopping baskets when they shop. Knowing which products are often shopped together helps retailers to formulate targeted marketing strategies. By exploring the associations between different products in the shopping process, interesting patterns in purchasing behavior can be uncovered, which in turn can help retailers to formulate appropriate marketing strategies.

Let $I=i_{1},i_{2},\ldots ,i_{3}$ be an itemset, where *m* is the number of items and $i_{k}\left(k=1,2,...,m\right)$ represents the *i*-th item. Transaction *T* represents a subset of set *I*, corresponding to individual orders. The set composed of transactions is denoted as $D(D=T_{1},T_{2},\ldots T_{n})$ and is called a transaction dataset. Usually, each transaction $T_{i}\left(i=1,2,\ldots n\right)$ has a unique number and contains several items. Association rules are implications in the form of X→Y(X∈I,Y∈I,X∩Y≠⌀), used to represent the implicit associations within data, where the strength of association rules is determined by support and confidence, defined as follows:

SupportX→Y indicates the probability of *X* and *Y* occurring together. The calculation formula is as follows:

(1) $$\mathrm{support}\left(X\to Y\right)=\frac{\left|T(X\cup Y)\right|}{\left|D\right|}$$

where |T(X∪Y)| represents the number of transactions containing both *X* and *Y*, and *D* represents the total number of transactions in the transaction dataset |D|. A minimum support min(supp) is usually set, and when SupportX→Y exceeds the min(supp), it is considered a frequent itemset. ConfidenceX→Y indicates the probability of *X* and *Y* occurring together given transaction *X* has occurred. The calculation formula is as follows:

(2) $$\mathrm{confidence}\left(X\to Y\right)=\frac{\left|T(X\cup Y)\right|}{\left|T(X)\right|}$$

where |T(X)| represents the number of transactions containing *X*.

The task of association rule mining can be divided into two distinct subtasks as follows: frequent pattern mining and the generation of association rules.

Frequent pattern mining [49] (also known as generating frequent itemsets) refers to screening frequent occurrences in a set of candidate items. Typically, the frequency of occurrence of each item is measured to determine whether it is a frequent item. If the occurrence count of an item surpasses a specified threshold, it can be regarded as a frequent item. The goal of this task is to find the set of items with high a frequency of occurrence in a large amount of data for further mining of association rules or application to other data mining tasks.

Generate association rules: In the generated frequent itemset, find association rules whose confidence is no less than the given minimum value confidence.

#### 3.1.3. Problem Definition

The activity recognition challenge can be interpreted as a classification task, with its primary goal being the assignment of activity labels to sequential events generated by a set of sensors. Each event consists of a sensor ID, a return value, and a timestamp. These events are arranged in chronological order to form a trajectory of activities, and the trajectory is associated with a specific activity label. An event is a value or state returned by a sensor when the sensor sends a signal as follows: $e_{i}=\left\{\begin{array}{c} s_{i},t_{i},\nu_{i} \end{array}\right\}$, where $t_{i}$ is the time at which the data were logged, $s_{i}$ is the id of the sensor, and $v_{i}$ is the value of the sensor. Then, there will be a large number of transactions over a period of time in the behavior. The sequence $L_{i}$ is a trace of the activity. Each $L_{i}$ can be associated with an activity label $a_{i}\in A$ through semantic segmentation.

### 3.2. Habit Mining Algorithm Architecture

**Figure 2:**
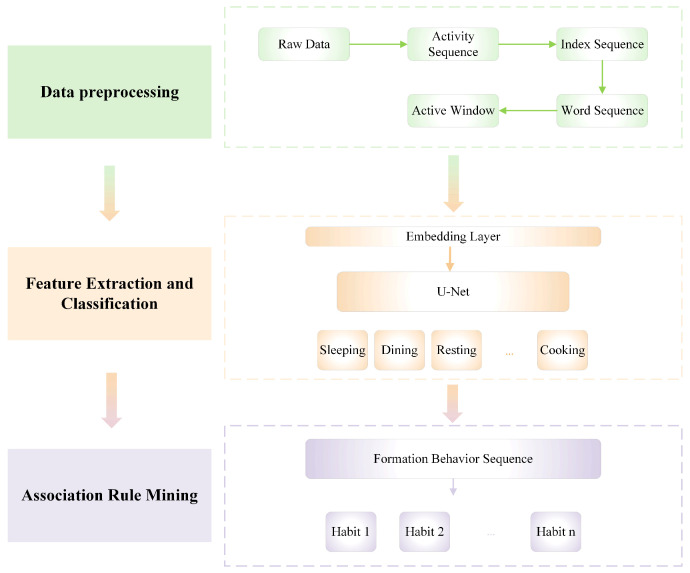
Framework architecture of the proposed method.

The habit mining algorithm combines a one-dimensional U-Net neural network and the FP-Growth algorithm to effectively classify and analyze behavior data. It utilizes U-Net for accurate activity recognition by processing sensor data and extracting relevant features. Subsequently, FP-Growth integrates temporal attributes to uncover time-sensitive associations and patterns in the behaviors, allowing the identification of meaningful habits of elderly individuals. This synergy enhances precision in behavior recognition and habit mining, particularly in smart home contexts. First, the raw data from the sensor are textualized and encoded into a series of indices. Then, a sliding window is used for splitting. Next, the sliding window is processed by embedding to extract the first-level features. After that, it is categorized by U-Net network. Finally, user habits are mined using an association relation algorithm. The algorithm framework architecture is shown in Figure 2.

The basic process of the habit mining algorithm for elderly living alone involves data collection, processing, feature extraction, model training, classification [50], and association rule analysis. Through this process, the user’s associated habits are mined from the original data.

#### 3.2.1. Data Preprocessing

In a smart home environment, sensor events are treated as text sentences, consecutively describing the behaviors of residents. Each behavior sequence is extracted and processed as a sentence in natural language processing (NLP), using the labels in the dataset to identify the start and end of each behavior.

The data generated by sensors is typically in the form of time-series, containing the sensor ID, return values, and timestamps. To effectively process these data and lay the foundation for subsequent behavior recognition and habit mining, we need to perform several key steps, as follows:

We treat sensor events as text sentences, where each event is composed of the sensor ID, its value, and the timestamp. For simplification, the timestamp is ignored, and only the sensor ID and its corresponding value are considered. For example, if the sensor M011 is “on” at a particular time, it is represented as “M011ON”. In this way, all sensor events form a text dataset, similar to a “vocabulary” in natural language processing, where each word (e.g., “M011ON”) represents a specific behavioral state.

To process these text data, we employ a frequency-based index embedding method [51], sorting these fields by frequency and assigning integer indices. This method transforms each sensor event into an index that can be used by neural networks. The indices are then converted into feature vectors through an embedding layer, which is used to map the sensor event indices to 64-dimensional vectors. Each sensor event is thus represented by a 64-dimensional vector. This dimensionality is set based on the typical word embedding dimensions used in NLP, allowing the network to learn internal representations of each “word” in every sensor event.

To handle behavior sequences of varying lengths, we apply a sliding window technique to segment the data into fixed-size windows. For windows with insufficient events, zero-padding is used to maintain a consistent window size.

Among the different windowing methods, the time window performed best in terms of accuracy and weighted F1 means, but was deficient in classifying certain behaviors. The sensor event window method was suboptimal in terms of accuracy and weighted F1 means, but it was able to classify other behaviors that the time window method could not. Although the dynamic window method only classifies individual behaviors, it matches the time window method on the F1 mean compared to the sensor event window method. Therefore, the sensor event window method is considered to be the best method for generating binary data for smart homes.

#### 3.2.2. Feature Extraction and Classification

**Figure 3:**
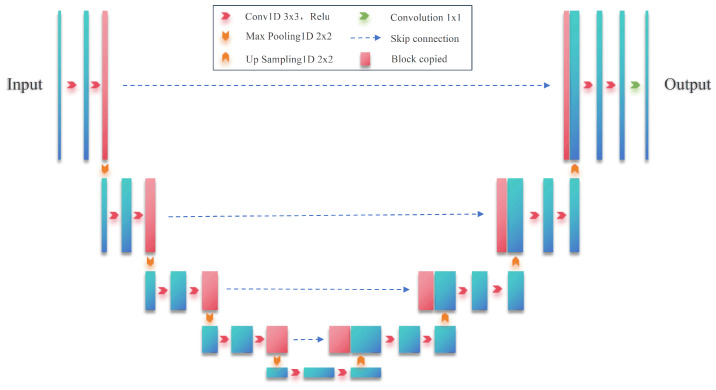
One-dimensional U-Net network structure diagram.

The processed data are passed through the U-Net network to produce behavioral sequence data. In the U-Net network architecture, the data are first convolved and pooled, for example, at the beginning of the length of the data is 64, and then, it becomes 32, 16, 8, and 4. Next, upsampling or deconvolution is applied to the feature data with a length of 8, resulting in feature data with a length of 16. This feature data are concatenated with the previously processed feature data of the same length (16) through channel-wise concatenation. The concatenated feature map is then further upsampled or deconvolved to produce feature data with a length of 32, which are subsequently concatenated with the earlier feature data of the same length (32). This process ultimately generates a prediction output that matches the size of the input data. The structure of the one-dimensional U-Net network is shown in Figure 3. The left half of Figure 3 is the encoder, which has three sub-modules, each of which contains two one-dimensional convolutional layers with a convolutional kernel of 3, and then, a downsampling layer is implemented by a maximal pooling layer with a step size of 2. The right half is the decoder, which consists of an upsampled convolutional layer, plus feature splicing concatenation and two convolutional layers with a convolutional kernel of 3, applied iteratively.

#### 3.2.3. Habit Mining Algorithm

After data processing, feature extraction, and classification, a series of behaviors are obtained. By applying the association rule algorithm to these behaviors, several groups of behavior rules can be derived. However, these behavior rules cannot be directly regarded as habits at this stage. In some behavior rules, such as $\left\{Behavior_{1},Behavior_{2},\ldots ,Behavior_{n}\right\}$, there may be a significant time interval between several behaviors. Although these behaviors are closely related in terms of frequency, the large time gaps between them means that they cannot be considered as human behavior habits.

**Figure 4:**
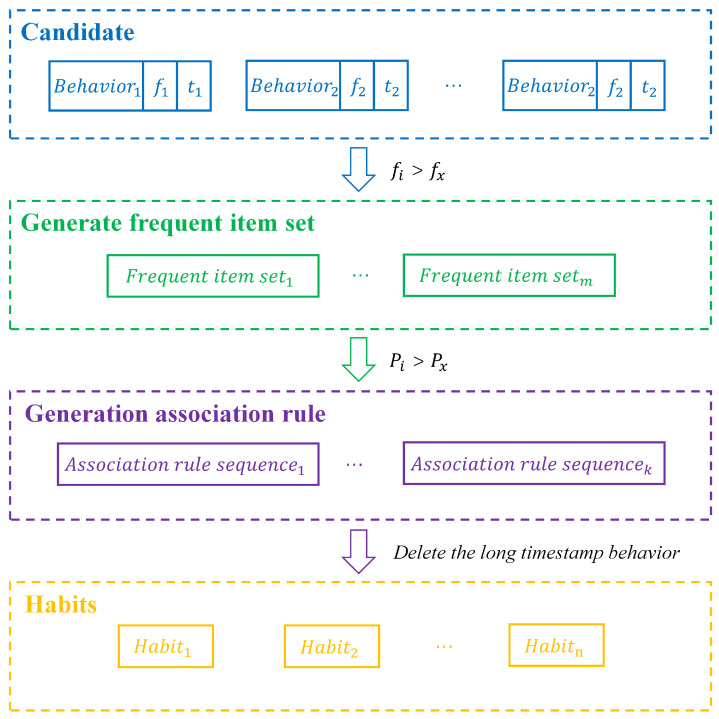
Flowchart of association rule analysis.

Therefore, in the process of generating associated behaviors, actions with large time gaps can be discarded based on their timestamps to address the above issue. Figure 4 represents the process of generating association rules for behaviors obtained after classification, where $Behavior_{i}$ denotes a certain behavior, $f_{i}$ denotes the number of times a behavior occurs, and $f_{x}$ denotes the set frequency threshold. $t_{i}$ denotes the time when the behavior occurs, i∈[1,n], and m∈[1,n]. $Frequentitemset_{j}$ represents the frequent itemset, and the behavior will only be included in the frequent itemset if its occurrence frequency is greater than the set threshold. $P_{j}$ denotes the confidence level of the frequent itemset and $P_{x}$ denotes the set confidence threshold, j∈[1,m]. $Associationrulesequence_{y}$ denotes the association rules generated after confidence filtering, k∈[1,m]. The generated association rules may include behaviors with long time intervals, which may not be relevant for mining users’ daily habits. Therefore, it is necessary to remove these behaviors with long timestamps in order to refine the rules to better align with daily habits. $Habit_{y}$ represents the set of association rules obtained after processing through the above steps, which align with daily habits and represent the user’s behavioral patterns.

## 4. Experiments

### 4.1. Experiments Setup

#### Experimental Data Preprocessing

The experiments were conducted using two major CASAS datasets, ARUBA and MILAN [52]. The CASAS dataset, developed by Washington State University, includes daily life data collected from real apartments and actual residents living in their own homes. The ARUBA dataset is small in size and has relatively simple activity types and scenarios. Since there is only one person living in the home, the activity data are more straightforward and there is no multi-user interference. The MILAN dataset is larger in size and higher in data complexity and contains data regarding the activities of multiple people at the same time. Activity recognition needs to deal with the cross-interference of different people’s activities, making the behavior recognition task more complex for the MILAN dataset. Temperature sensors and binary sensors, etc., are installed in the house to monitor the behavior or door opening and closing, etc.

### 4.2. Evaluation Metrics

The algorithm proposed in this paper divides the dataset into two parts as follows: 70% for training and the remaining 30% for testing. These are randomly disrupted to contain a variety of behaviors, and the number of behaviors is proportionally distributed. The layered design ensures that the two subsets cover a variety of behavior types. In this paper, we perform a layered triple cross-validation process.

Next, the algorithm is trained and validated using these three training subsets and three validation subsets. Early stopping and optimal model selection strategies, proposed by the Tensorflow framework, were used in each training phase.

The use of this strategy makes the model stop training as soon as overfitting occurs and then saves the optimal model. The strategy is based on verifying the loss value. If the loss value of the current training is consistently lower than the loss value of the current optimal model after more than n epochs, the training will stop and proceed to the next step. In addition, during testing, it was found that different batch sizes had no significant effect on the evaluation results, which were similar except for the training time.

Sequential results of generated behaviors are used as a dataset to mine habits by association rule algorithms, and the algorithm performance is evaluated using the precision metric.

(3) $$\mathrm{Precision}=\frac{\mathrm{True}\mathrm{Positives}(\mathrm{TP})}{\mathrm{True}\mathrm{Positives}(\mathrm{TP})+\mathrm{False}\mathrm{Positives}(\mathrm{FP})}$$

#### Implementation Settings

The operating system used in the experimental environment of this paper is Windows 10, the CPU is AMD processor Ryzen 7 4800H, the GPU is NVIDIA GTX1650, the RAM is 16G, and the video memory is 4G. (NVIDIA GeForce GTX 1650 graphics card is manufactured by NVIDIA in Taiwan, China, and AMD Ryzen 7 4000 series processors are manufactured by TSMC in Taiwan, China. Ram and Video manufacturers are Ramaxel in Shenzhen, China) In addition, the algorithms of this paper use the programming language Python, version 3.8.

The sequence of events is split in SEW. Different SEW sizes were investigated, 100, 75, 50, 25, with a step size of 1. This step size allows the HAR process to be performed each time a new event is triggered. The goal is to find the optimal SEW size, e.g., the smallest SEW size with maximum information that allows the differentiation of the activity sequences with high F1 scores and high balanced accuracy. The smaller the size of the SEW, the faster the recognition of the activity.

All the convolutional layers in FCN have a step size of 1 and a zero padding strategy is used to maintain the original length of the time series after convolution. The first convolutional layer contains 128 filters with 1D convolution, i.e., kernel size 8, followed by a second convolutional layer containing 256 filters with 1D convolution, i.e., kernel size 5. These filters are further fed into a third convolutional layer consisting of 128 filters with 1D convolution, i.e., kernel size 3. In terms of the LSTM, the network consists of 64 neurons, followed immediately by the final classification for the softmax layer. Since LSTM was originally designed for the field of NLP (Natural Language Processing), an embedding layer is incorporated between the raw data and the neural network, with the number of neurons set to 64, as in the literature [53]. The step size of all convolutional layers in the U-Net is also 1, in zero-padding mode, and the filter parameters of each convolutional layer of the encoding layer are 64, 128, 256, 512, and 1024, in order, with the convolutional kernel set to 3 and the pooling layer for each downsampling being a maxpool with a parameter set to 2. The parameters of each convolutional layer of the decoding layer are 512, 256, 128, and 64, and the size of the one-dimensional convolutional kernel is also configured to 1. The size of the one-dimensional kernel is also set to 1. The size of the one-dimensional convolutional kernel is likewise set to 3, while the upsampling parameter is set to 2.

By setting different minimum support levels and different minimum confidence levels, we form parameter combinations in pairs without repetition, and we then conduct experiments with different parameter combinations. The range of values of minimum support is set to be from 0.75 to 0.85, and 20 values are taken on average. The minimum confidence level is set to be from 0.75 to 0.85, and 20 values are taken on average.

### 4.3. Ablation Experiments and Sensitivity Analysis

**Figure 5:**
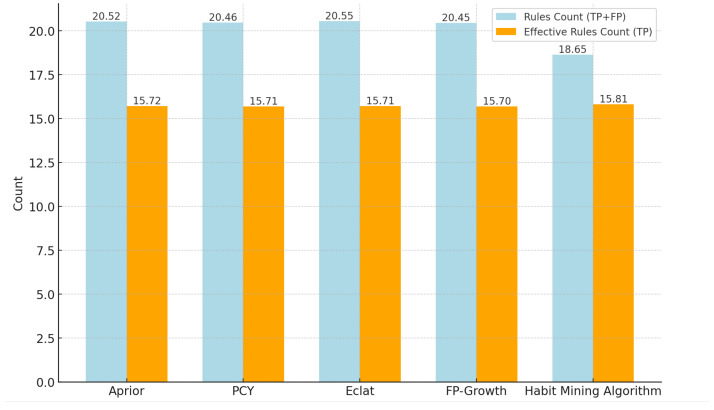
The total number of rules and the number of valid rules of the algorithm.

**Figure 6:**
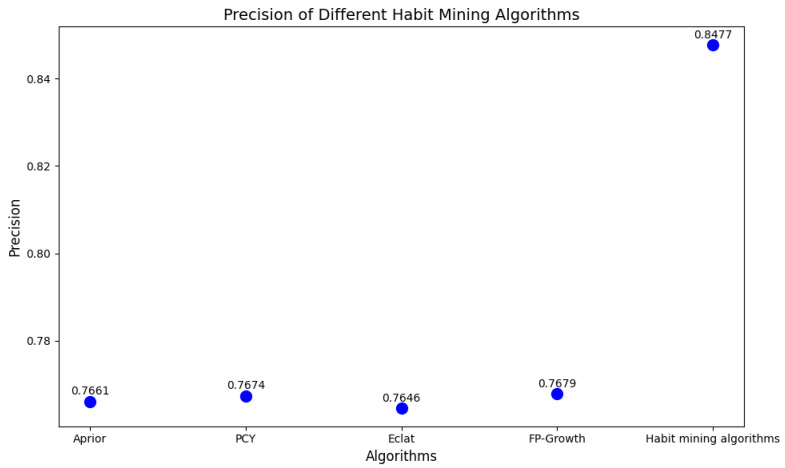
Scatter plot: The value of algorithm-based precision.

Some experiments were implemented on the model presented in this paper under the two major datasets of CASAS, ARUBA and MILAN; the parameters were adjusted; and the final experimental results are shown in Figure 5 and Figure 6.

Table 1, Table 2, Table 3 and Table 4 show the performance of FCN, LSTM, and U-Net on raw sensor data from both datasets. LSTM, FCN, U-Net, and normal LSTM, FCN (SOTA), and U-Net with embedding layers added on different window sizes are evaluated. The average balanced accuracy and average weighted F1 value are calculated.

Table 1 and Table 2 show that the weighted F1 mean of the LSTM can only approach the FCN at a larger SEW in order to have its normal effect, indicating that a larger number of events are needed for training when applying the LSTM. In addition, U-Net obtains the highest weighted F1 mean with and without embedding both datasets, and this paper suggests that this may be due to the multilayer downsampled convolution and skip connection design of the U-Net network, which allows for the retention of important features in high-level convolution.

It can be observed from Table 3 and Table 4 that U-Net has the highest average balancing accuracy, except on the MILAN dataset with a window of 100. The performance of the convolutional neural network decreases in large windows, probably because large windows are not conducive to the retention of behavioral sequence features for small numbers of events. In large windows, some small sequences, such as $Leave_{H}om$, are left unclassified, which can lead to a decrease in the balanced accuracy score. In contrast, U-Net can obtain the highest average balancing accuracy in the small window, while the weighted F1 mean also obtains a high score.

Meanwhile, from the data in these tables, it can be seen that putting aside the effect of SEW on each network, the performance of the network follows the order U-Net, FCN, and LSTM. Meanwhile, the addition of an embedding layer significantly improves the performance of the network. After adding the embedding layer, the LSTM algorithm showed a significant improvement in average balanced accuracy, which may be related to the frequency encoding strategy. Frequency encoding helps LSTM focus on rarer or more significant events, particularly when SEW is large. In such cases, LSTM can better leverage these encodings to identify key events, which explains why LSTM achieves the highest F1 score with larger SEW.

Behavior recognition can be well classified and the data will be processed for correlation relationship analysis by setting different combinations of parameters of minimum support and minimum confidence. We repeat the experiment according to the experimental setup and take the mean value of the results of each index, and the final experimental results are shown in Figure 5 and Figure 6.

From Figure 5 and Figure 6, it can be seen that the number of rules mined by each association rule algorithm from frequent itemsets is generally consistent. This suggests that each algorithm is similarly effective at mining the user’s behavioral association patterns.The number of rules (18.653) mined by the habit mining algorithm with time-sensitive parameter processing is less than that mined by the above algorithms, while the number of effective rules has not decreased. This indicates that the algorithm will exclude the rules that do not meet the requirements. After comparison, it is exactly the rules that do not conform to the rules to be mined in the environment described in this paper that are excluded. The experimental results show that the traditional association rule algorithms have approximately the same effect, but none of them consider the impact of time series on mining behavior association rules. The experimental analysis demonstrates that the proposed approach is not only efficient but also exhibits superior performance, and it can more accurately discover the potential time-series related behavior habits of users.

In an effort to evaluate the impact of each module in the proposed algorithm on overall performance, we conducted ablation experiments. The effectiveness of each module is analyzed by gradually removing or replacing specific modules and observing the performance changes. The experimental results are shown in Table 5.

The experimental results indicate that when using only the FP-Growth(sota) algorithm, although a certain number of association rules can be mined (total number of rules is 20.450, with 15.703 valid rules), the precision is only 0.7679. This suggests that the accuracy of the mining results is relatively low. The reason is that directly performing association rule mining on raw data is susceptible to interference from noisy data and irrelevant information, thereby affecting mining effectiveness.

In contrast, when combining the U-Net and FP-Growth algorithms, the total number of mined rules slightly decreases to 19.974, but the number of valid rules increases to 15.774, and the precision improves to 0.7897. This indicates that the introduction of U-Net effectively filters out some noisy data during the data processing stage, enhancing the accuracy of rule mining.

Furthermore, by integrating embedding techniques with U-Net and FP-Growth, the total number of rules further decreases to 18.653, while the number of valid rules increases to 15.813, and the precision significantly improves to 0.8477. This demonstrates that the incorporation of embedding techniques can further improve data representation, significantly enhancing the effectiveness of subsequent association rule mining.

In summary, the complete algorithm (U-Net + Embedding + FP-Growth) fully leverages the advantages of the U-Net network and embedding techniques during the data preprocessing and behavior classification stages. It effectively removes noisy data and provides more accurate foundational data for subsequent association rule mining, thereby significantly improving the overall performance of the algorithm.

## 5. Related Discussion

### 5.1. Discussion on Superiority

Compared to traditional association rule algorithms (such as Apriori), FP-Growth offers higher computational efficiency when handling large datasets and does not require generating candidate itemsets, thereby reducing computational complexity. The traditional Apriori algorithm necessitates multiple scans of the database, which can be inefficient when processing complex and large sensor data. In contrast, FP-Growth enhances efficiency by constructing a compressed FP-tree.

Although Long Short-Term Memory (LSTM) networks are capable of processing time-series data, LSTM models exhibit higher computational complexity, longer training times, and poorer performance in capturing short-term dependencies. In contrast, the 1D U-Net excels at handling short-term dependencies, making it particularly suitable for capturing and recognizing local features in sensor data, offering superior computational efficiency and real-time performance.

The combination of 1D U-Net and FP-Growth fully leverages the strengths of both approaches. The U-Net is capable of extracting temporal dependency features from the input data, while FP-Growth can automatically mine frequent behavioral patterns and association rules based on these features. Compared to using other traditional machine learning methods or deep learning models individually, this combination achieves higher recognition accuracy and lower computational costs.

### 5.2. Discussion on Limitation

In terms of algorithm design, in real-world environments, the algorithm faces data from different scenarios, and the parameters set in this study may not effectively adapt to the impact of scene-specific differences. Additionally, sensor data in real-life scenarios are often susceptible to noise interference, such as electromagnetic disturbances and sensor malfunctions. Since the experiments did not specifically test for noise interference, the performance of the algorithm in high-noise environments remains unclear. From the perspective of scalability in large-scale data scenarios, with the widespread adoption of smart home devices, the volume of data will grow rapidly. The dataset used in the current study is relatively small, and when processing large-scale data, the computational load and memory requirements of the FP-Growth algorithm may increase significantly, while the training time of the U-Net network may also be greatly extended, which limits the algorithm’s application in large-scale data environments.

Regarding sensors, considering that the data collected by sensors may contain sensitive information, there is a risk of privacy breaches due to the collection of personal data from elderly individuals. Moreover, the reliability of sensors cannot be overlooked, as environmental factors, battery life, and other issues may lead to inaccurate or lost sensor data, affecting system performance. Therefore, it is essential to choose highly reliable sensors, perform regular maintenance and calibration, or develop new low-power, self-calibrating sensors.

## 6. Conclusions

This paper proposes a novel AIoT-based habit mining algorithm for elderly individuals living alone. The algorithm integrates an embedding layer with a U-Net model for behavior recognition and employs the FP-Growth algorithm for habit mining. Experimental validation on the CASAS datasets (ARUBA and MILAN) demonstrates the effectiveness of the proposed algorithm. Specifically, the combination of U-Net and the embedding layer achieves an average weighted F1 score of 100% on the ARUBA dataset and 99% on the MILAN dataset, highlighting its high accuracy. In terms of association rules, the proposed algorithm shows a 7.98% improvement in precision compared to the existing state-of-the-art (sota) algorithm.

In the real-world smart home environment, through accurate behavior classification and the analysis of temporal association rules, this algorithm can effectively identify the long-term living habits of the elderly, providing an efficient and highly adaptable monitoring framework for smart home systems.In terms of enhancing the quality of life of the elderly, this algorithm enables family members and caregivers to have a better understanding of the daily behavior patterns of the elderly, thereby facilitating the provision of more personalized care services.Regarding safety monitoring, if the elderly engage in activities that deviate from their usual habits, such as staying outside the bedroom for an extended period after getting up at night or exhibiting abnormal behavior in high-risk areas like the bathroom, corresponding rules can be designed to notify relatives. This can effectively prevent the occurrence of accidents and improve the living safety of the elderly.

Future work will focus on optimizing the algorithm by exploring better network architectures and adjusting association rule parameters to improve algorithm performance. In addition, efforts will be concentrated on addressing the aforementioned challenges, including the development of data privacy protection technologies, methods to enhance sensor reliability, and the enhancement of the system’s adaptability to diverse living conditions. For example, the system will be tested and optimized in more diverse scenarios, and personalized configuration functions will be developed. By addressing these issues, it is expected that the application of this algorithm in the fields of smart homes and elderly care can be further expanded, thereby improving the safety and quality of life for elderly individuals living alone. 

## Figures and Tables

**Table 1:** Weighted F1 score in ARUBA’s dataset.

Model	25	50	75	100
LSTM	87.00	90.67	93.67	97.00
FCN	91.00	97.33	97.33	98.67
U-Net	95.00	97.33	97.33	99.33
LSTM+Embedding	92.00	98.00	98.00	100.00
FCN+Embedding	99.67	99.67	100.00	100.00
**U-Net+Embedding**	**99.67**	**100.00**	**100.00**	**100.00**

**Table 2:** Weighted F1 score in MILAN’s dataset.

model	25	50	75	100
LSTM	68.00	75.00	84.67	84.00
FCN	83.00	88.67	91.33	76.00
U-Net	93.33	93.00	94.00	95.00
LSTM+Embedding	75.33	93.33	96.67	96.67
FCN+Embedding	94.00	96.67	96.67	98.33
**U-Net+Embedding**	**95.00**	**97.33**	**98.00**	**99.00**

**Table 3:** Balanced accuracy in ARUBA’s dataset.

Model	25	50	75	100
LSTM	81.45	70.37	77.68	80.33
FCN	83.42	85.68	85.07	85.79
U-Net	90.43	91.07	90.20	91.61
LSTM+Embedding	76.34	89.77	94.23	95.08
FCN+Embedding	93.27	95.07	95.76	95.87
**U-Net+Embedding**	**95.23**	**95.89**	**96.03**	**95.91**

**Table 4:** Balanced accuracy in MILAN’s dataset.

Model	25	50	75	100
LSTM	48.67	57.70	62.71	60.34
FCN	74.91	75.15	77.65	67.97
U-Net	82.77	83.30	84.15	83.57
LSTM+Embedding	61.35	82.05	84.57	87.93
FCN+Embedding	90.30	87.83	86.77	85.57
**U-Net+Embedding**	**92.15**	**89.86**	**88.25**	**84.92**

**Table 5:** Balanced accuracy in MILAN’s dataset.

Experimental Setup	Number of Rules (TP+FP)	Number of Valid Rules (TP)	Precision
U-net only	/	/	/
FP-Growth	20.450	15.703	0.7679
U-Net+FP-Growth	19.974	15.774	0.7897
**U-Net+Embedding+FP-Growth**	**18.653**	**15.813**	**0.8477**

## Data Availability

The results of this study are available from the corresponding author upon reasonable request.
